# Outcomes of Total Hip Arthroplasty in Patients With Acetabular Protrusio

**DOI:** 10.5435/JAAOSGlobal-D-20-00121

**Published:** 2020-07-20

**Authors:** Danielle Greig, Peter P. Hsiue, Clark J. Chen, Rishi Trikha, Amir Khoshbin, Alexandra I. Stavrakis

**Affiliations:** From the Department of Orthopaedic Surgery, David Geffen School of Medicine at UCLA, Los Angeles, CA (Dr. Greig, Dr. Hsiue, Mr. Chen, Dr. Trikha, and Dr. Stavrakis), and the Division of Orthopaedic Surgery, University of Toronto, Toronto, Ontario (Dr. Khoshbin), Canada.

## Abstract

**Background:**

Acetabular protrusio (AP) is associated with distorted anatomic landmarks and insufficient bone stock that increases complexity of total hip arthroplasty (THA). This study used a large national database to compare outcomes after THA in patients with and without AP.

**Methods:**

The Nationwide Readmissions Database was used to identify patients with and without AP who underwent THA from 2010 to 2014. Primary outcomes analyzed included complications during index hospitalization and within 90 days of THA.

**Results:**

Propensity score matching generated 4,395 patients without AP and 4,603 patients with AP. Patients with AP were older (68.1 versus 65.2 years, *P* < 0.0001), more predominantly women (82.1% versus 55.9%), and had more medical comorbidities as measured by the Elixhauser Comorbidity Index (2.29 versus 1.89, *P* < 0.0001). Patients with AP had an increased risk of requiring bone graft (odds ratio [OR] = 47.97, 95% confidence interval [CI]: 14.27 to 161.22), receiving a blood transfusion (OR = 1.90, 95% CI: 1.57 to 2.29), and suffering a periprosthetic fracture (OR = 2.56, 95% CI: 1.10 to 5.97) within 90 days of THA. Length and cost of index hospitalization were greater for patients with AP (5.0 versus 4.3 days, *P* = 0.002; $19,211.88 versus $27,736.30, *P* < 0.0001).

**Conclusion:**

Given the current emphasis on hospital cost optimization, it is important to ensure that patients with AP are managed appropriately. Attention should be placed on comprehensive preoperative planning and postoperative monitoring in this population.

Acetabular protrusio (AP) is defined as an acetabular defect causing medial, intrapelvic displacement of the acetabulum and femoral head^1,2,3,4^ (Figure 1, A). The etiologies of AP are vast because AP can stem from a primary idiopathic disorder or from secondary causes including trauma, malignancy, Paget disease, and inflammatory conditions such as rheumatoid arthritis (RA) and psoriatic arthritis.^4,5,6^ Furthermore, the overall prevalence of AP remains unknown^7^ because it varies across specific populations. For example, 27% to 31% of patients with Marfan syndrome and 5% of patients with RA develop AP.^8,9,10^ AP is also seen in patients with end-stage osteoarthritis. Patients with AP often present with notable pain and loss of function for which they desire surgical intervention.^5,6^ Although surgical options such as arthrodesis, resection arthroplasty, and valgus intertrochanteric osteotomy have been used historically, total hip arthroplasty (THA) is currently the preferred surgical treatment in this patient population^7,11,12^ (Figure 1, B).

**Figure 1:**
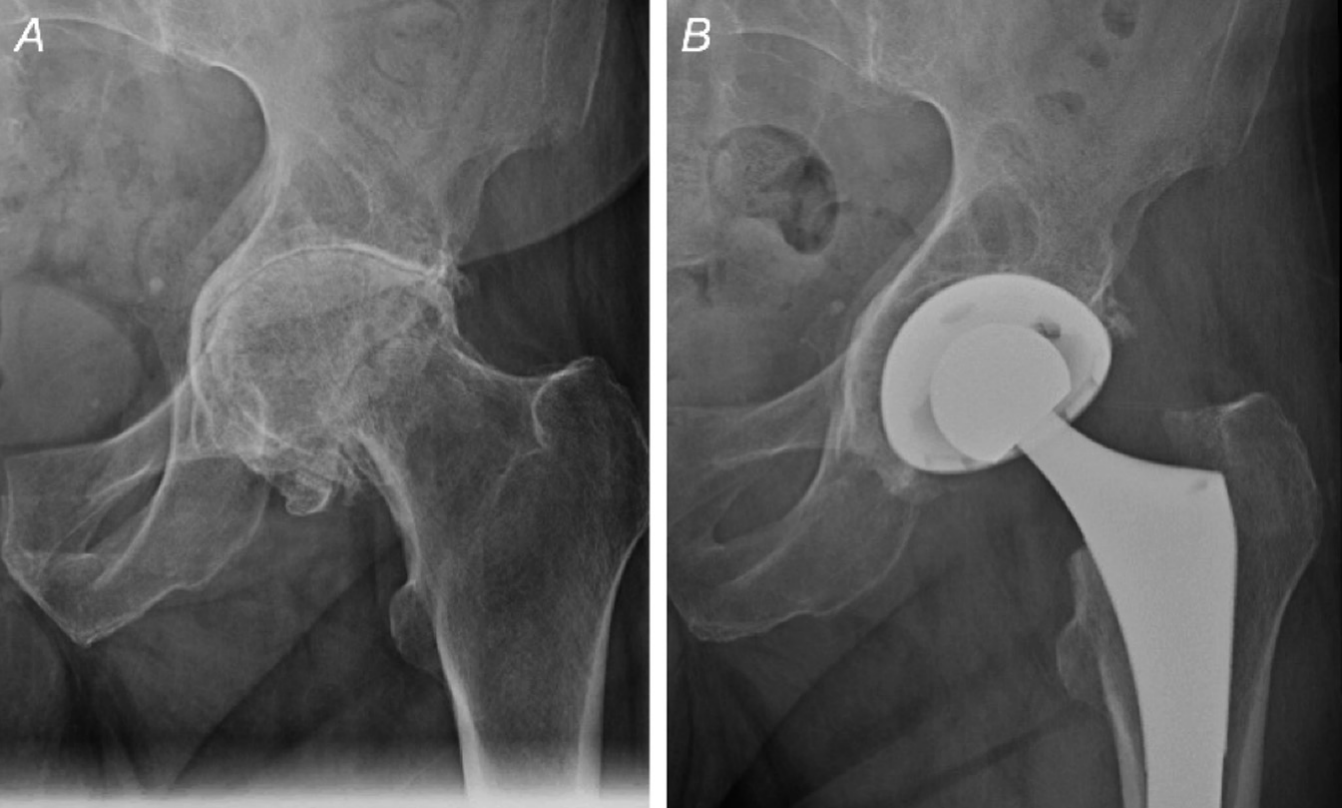
**A** and **B**, Radiographs of the hip demonstrating acetabular protrusio (**A**) in a patient who was treated with total hip arthroplasty (**B**).

Previous literature has demonstrated satisfactory short- and long-term outcomes for patients with AP who undergo THA.^11,12,13,14,15,16^ However, a higher level of anatomical complexity associated with these cases exists, including insufficient acetabular bone stock and loss of standard surgical landmarks. This can lead to improper acetabular component placement and potentially increased rates of aseptic loosening, instability, and the need for revision surgery.^17,18,19^ These intraoperative difficulties, along with the medical comorbidities not infrequently seen in this patient population, can make preoperative planning and postoperative care challenging for patients with AP.

To date, most studies of AP have included a relatively small number of patients (<100), have only analyzed a certain subset of patients with AP (eg, patients with Marfan syndrome or RA), or have only focused on a specific portion of the surgical technique such as the use of morselized bone grafting.^9,10,11,14,17^ The objective of this study was to utilize a large, national database to compare outcomes after THA in patients with AP to patients without AP. Furthermore, this study aimed to quantify differences in healthcare resource utilization between these two groups. It was hypothesized that patients with AP who underwent THA would have worse overall outcomes than patients without AP, with a higher risk of mortality and postoperative complications. It was also hypothesized that these patients would have increased healthcare resource utilization during index hospitalization, specifically longer length of stay (LOS) and higher cost of stay.

## Methods

The Nationwide Readmissions Database (NRD) was used to identify the study cohort of interest over a 5-year period (2010 to 2014).^20^ The NRD is a database that compiles inpatient data from 22 states and tracks patients across multiple hospitals. This database is validated by a federal-state-industry partnership sponsored by the Agency for Healthcare Research and Quality. Approximately 51.2% of the total US population and 49.3% of all US hospitalizations were sampled in a stratified algorithm, allowing for an accurate estimation of nationally representative statistics.^20^ Available variables include patient demographic data, diagnoses, procedures, hospital charges, hospital LOS, and hospital characteristics. The deidentification of the NRD deemed this study exempt from our institution's Institutional Review Board.

All patients older than 18 years of age who underwent THA were included for analysis. Patients were selected using the International Classification of Diseases, Ninth Revision, (ICD-9) procedure code for THA (81.51). This cohort of patients was then subdivided into 2 groups that were analyzed against one another, those with AP and those without AP at the time of THA. These subsets were identified using the ICD-9 diagnosis code for AP (718.65) (Figure 1 and Table 1). The Elixhauser Comorbidity Index (ECI), a composite score of 31 comorbid conditions, was used to quantify baseline comorbidity, with higher scores corresponding to a greater burden of comorbid conditions.^21^

**Table 1:** ICD-9 Codes Used for Identifying Patients of Interest

Diagnosis	ICD-9 code
Acetabular protrusio	718.65
Rheumatoid arthritis	714.0
Procedures	ICD-9 code
Total hip arthroplasty	81.51
Bone graft	78.0, 78.01-09

ICD-9 = International Classification of Disease, 9th Revision

**Figure 2:**
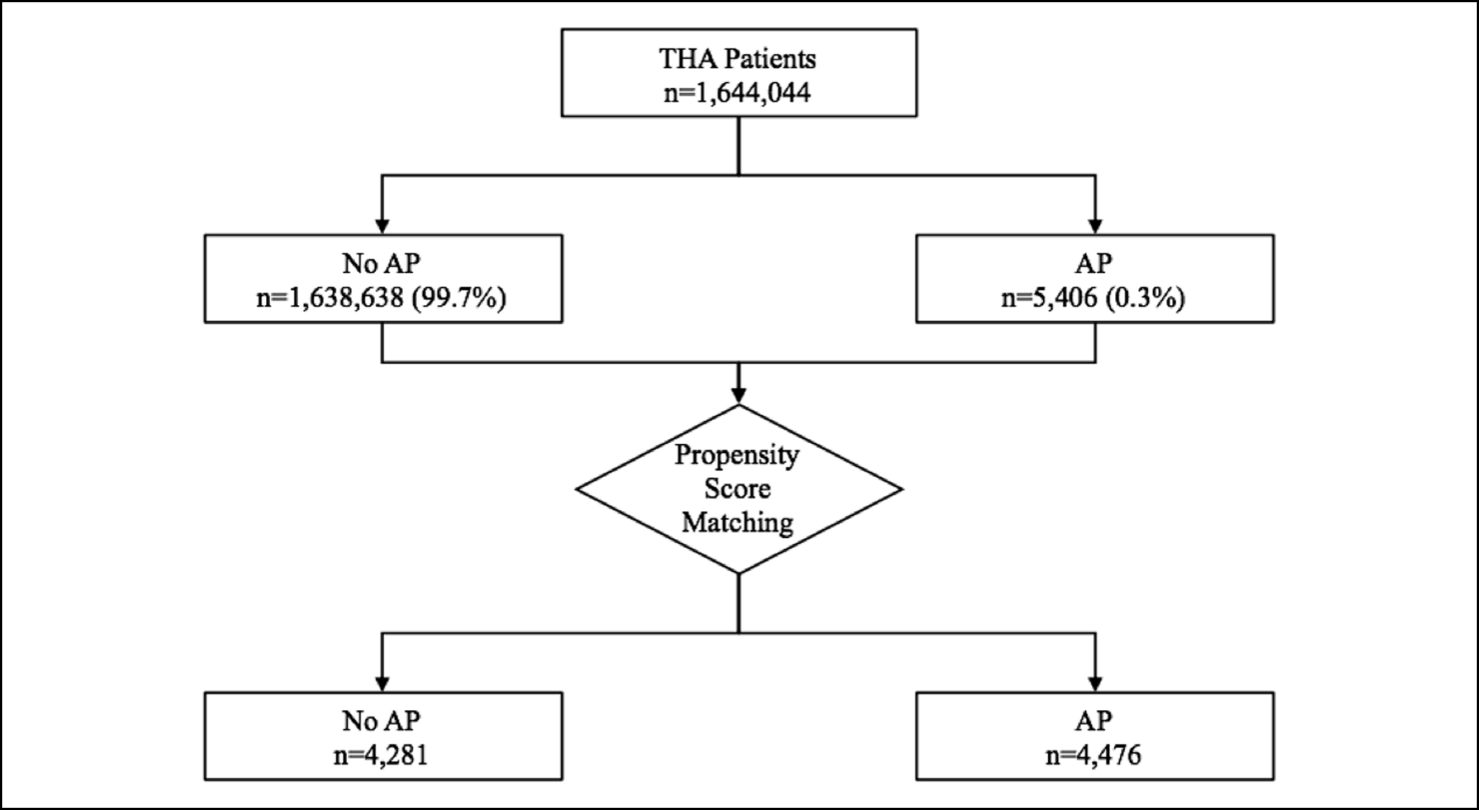
THA patients were divided into two groups based on diagnosis of AP. One-to-one propensity matching was done to create two matched cohorts for comparison and analysis. AP = acetabular protrusio, THA = total hip arthroplasty

Propensity score matching (PSM) was done to account for baseline differences between the cohort with AP and the cohort without AP (Figure 2). Calipers were set to a width of 0.2 and matching was done using the nearest neighbor without replacement method.^22,23^ A propensity score was set using a logistic regression model with covariables selected from patient demographics (age, sex, insurance type, and income), comorbidities (ECI), a diagnosis of RA (ICD-9 718.65) (Table 1), and provider parameters (hospital size and type). Primary and secondary outcomes were then analyzed postmatching.

The primary outcomes of interest included the need for bone grafting intraoperatively, need for blood transfusion, discharge location, mortality, and postoperative complications during the index hospitalization and within 90 days of THA. Postoperative complications during the index hospitalization that were evaluated included cardiac and respiratory complications, deep vein thrombosis, pulmonary embolism, acute renal failure, surgical site infection, systemic infection, and wound complications. Postoperative complications after hospital discharge and within 90 days of surgery included hospital readmission, postoperative dislocation, acetabular or femoral periprosthetic fracture, revision surgery for any reason, revision surgery for instability, revision surgery for periprosthetic fracture, and revision surgery for infection. ICD-9 codes were used to identify patients with postoperative dislocation (996.42), periprosthetic fracture (996.44), and revision surgery (81.53, 80.05, 80.15, 00.70, 00.71, 00.72, and 00.73). Secondary outcomes included index hospitalization LOS and cost of stay as well as discharge location (home versus skilled nursing facility) after the index hospitalization. Individual cost was calculated by multiplying diagnosis-related group codes by hospital-specific cost-to-charge ratios provided by the Agency for Healthcare Research and Quality and subsequently adjusting for inflation using the respective yearly gross domestic product.^24^ These estimates were further adjusted for via the use of the Healthcare Cost and Utilization Project indices of the diagnosis-related group to account for differences between hospitals. ICD-9 codes used for identifying postoperative complications are listed in Appendix 1 (http://links.lww.com/JG9/A82).

All result sample sizes represented national annual estimates, accounting for individual discharge-level weights from the NRD's stratified two-stage cluster design. Descriptive analysis was used to describe baseline characteristics and outcomes for each comparison group. Continuous variables were reported as means, 95% confidence intervals (CIs) and *P*-values. Categorical variables were reported as adjusted odds ratios (ORs) and compared using a Chi-square test, except when there were less than 10 individuals in a subset, in which a Fisher exact test was used. After ensuring normal distributions, analysis was done using a two-tailed Student *t*-test. For skewed distributions, continuous variables are presented as a median and analyzed using the Wilcoxon rank-sum test. All data were stored and analyzed using Stata 15.1 Statistical analysis was done by comparing patients with AP who underwent THA with patients without AP who underwent THA. All tests were set at a significance level defined at *P* < 0.05.

## Results

A total of 1,644,044 patients who underwent THA from 2010 to 2014 were identified, of which 5,406 (0.3%) had a diagnosis of AP and 1,638,638 (99.7%) did not (Figure 1). PSM generated 4,395 patients with no diagnosis of AP and 4,603 patients with a diagnosis of AP for comparative analysis (Figure 1).

### Patient Characteristics

As compared to patients without AP who underwent THA, patients with AP were found to be older (68.1 versus 65.2 years, *P* < 0.0001), more predominantly women (82.1% versus 55.9%, *P* < 0.0001), and have more medical comorbidities as measured by ECI (2.29 versus 1.89, *P* < 0.0001) (Tables 2 and 3). In addition, patients with AP were more likely to be insured under Medicare (64.8% versus 53.6%, *P* < 0.0001) and less likely to have private insurance (27.9% versus 39.0%, *P* < 0.0001). Regarding hospital size, patients with AP were more likely to be treated at a large hospital (59.8% versus 55.7%, *P* = 0.011). No notable difference was noted in hospital type detected between the 3 groups (urban teaching, urban nonteaching, and rural) (Table 3). PSM results were examined, and no notable difference remained regarding age, sex distribution, insurance status, hospital size, or hospital type (Table 4).

**Table 2:** Patient Age and ECI

	Age (Years)			ECI		
	Mean	95% CI	*P* value^a^	Mean	95% CI	*P* value^a^
No protrusio	65.2	65.1-65.3	—	1.89	1.87-1.91	—
Protrusio	68.1	67.3-68.8	<0.0001	2.29	2.20-2.37	<0.0001

CI, confidence interval; ECI, Elixhauser Comorbidity Index

a *P*-value when compared with no protrusio group

**Table 3:** Patient Demographics

	No Protrusio		Protrusio	
	n (%)	*P* value^a^	n (%)	*P* value^a^
Sex				
Male	**722,512 (44.1)**	**—**	**970 (17.9)**	**<0.0001**
Female	**916,126 (55.9)**	**—**	**4,436 (82.1)**	**<0.0001**
Insurance				
Medicare	**877,702 (53.6)**	**—**	**3501 (64.8)**	**<0.0001**
Medicaid	59,825 (3.7)	—	222 (4.1)	0.3349
Private	**638,915 (39.0)**	**—**	**1,507 (27.9)**	**<0.0001**
Self	10,830 (0.6)	—	27 (0.5)	0.3048
Hospital size				
Small	**340,515 (20.8)**	**—**	**898 (16.6)**	**0.0027**
Medium	385,965 (23.6)	—	1,275 (23.6)	0.9784
Large	**912,158 (55.7)**	**—**	**3,233 (59.8)**	**0.011**
Hospital type				
Urban teaching	901,224 (55.0)	—	3,115 (57.6)	0.2576
Urban nonteaching	593022 (36.2)	—	1,869 (34.6)	0.2576
Rural	144,392 (8.8)	—	422 (7.8)	0.348

a *P*-value when compared to no Protrusio group. Statistically significant (p<0.05) for bolded entries.

**Table 4:** Patient Characteristics in Propensity Score Matched Cohorts

	Age (Years)			Elixhauser Comorbidity Index		
	Mean	95% CI	*P* value^a^	Mean	95% CI	*P* value^a^
No protrusio	67.7	67.0-68.4	—	2.37	2.28-2.47	—
Protrusio	67.5	66.7-68.3	0.6862	2.37	2.28-2.47	0.9702

	No Protrusio		Protrusio	
	n (%)	*P* value^a^	n (%)	*P* value^a^
Sex				
Male	915 (20.8)	—	929 (20.2)	0.6806
Female	3,480 (79.1)	—	3,674 (79.8)	0.6806
Insurance				
Medicare	2,736 (62.2)	—	2,903 (63.1)	0.6623
Medicaid	179 (4.1)	—	197 (4.3)	0.7715
Private	1,291 (29.4)	—	1,332 (29.0)	0.8026
Self	27 (0.6)	—	27 (0.6)	0.8946
Hospital size				
Small	775 (17.6)	—	843 (18.3)	0.711
Medium	1,053 (24.0)	—	1,133 (24.6)	0.713
Large	2,567 (58.4)	—	2,627 (57.1)	0.5206
Hospital type				
Urban teaching	2,486 (56.6)	—	2,595 (56.4)	0.9244
Urban nonteaching	1,617 (36.8)	—	1,608 (34.9)	0.3053
Rural	292 (6.6)	—	400 (8.7)	0.0662

CI = confidence interval

a *P*-value when compared to No protrusio group.

### Clinical Outcomes and Healthcare Resource Utilization

After matching for ECI and RA, patients with AP were found to be more likely to require bone grafting intraoperatively (6.5% versus 0.1%, OR = 47.97, 95% CI: 14.27 to 161.22, *P* < 0.0001) and require blood transfusions (28.5% versus 17.3%, OR = 1.90, 95% CI: 1.57 to 2.29, *P* < 0.0001) during the index hospitalization (Table 5). No notable difference in mortality was detected between the 2 groups, although patients with AP had higher rates of cardiac complications (43.4% versus 39.1%, OR = 1.19, 95% CI: 1.01 to 1.41, *P* = 0.034) during the index hospitalization. Patients with AP were also more likely to be discharged to skilled nursing facility (42.3% versus 32.4%, OR = 1.53, 95% CI: 1.30 to 1.79, *P* < 0.0001).

**Table 5:** Clinical Outcomes

	Index Hospitalization				
	No Protrusio n (%)	Protrusio n (%)	OR	95% CI	*P* value
Mortality	2 (0.04)	11 (0.2)	6.20	0.71-53.85	0.098
Bone graft	**6 (0.1)**	**298 (6.5)**	**47.97**	**14.27-161.22**	**<0.0001**
Blood transfusion	**761 (17.3)**	**1,310 (28.5)**	**1.90**	**1.57-2.29**	**<0.0001**
Complications					
Cardiac	**1,719 (39.1)**	**1,998 (43.4)**	**1.19**	**1.01-1.41**	**0.034**
Respiratory	118 (2.7)	119 (2.6)	0.96	0.57-1.62	0.5703
Pulmonary embolism	6 (0.1)	22 (0.5)	3.34	0.90-12.45	0.8956
Deep vein thrombosis^a^	—	—	—	—	—
Acute renal failure	139 (3.2)	214 (4.7)	1.49	0.98-2.28	0.9798
Surgical site complication	43 (1.0)	38 (0.8)	0.85	0.37-1.92	0.3727
Systemic complication	20 (0.4)	23 (0.5)	1.11	0.33-3.76	0.3276
Wound complication	34 (0.7)	55 (1.2)	1.57	0.75-3.30	0.7454
Discharge					
Home	**2,941 (66.9)**	**2,594 (56.3)**	**0.64**	**0.54-0.75**	**<0.0001**
Skilled nursing facility	**1,424 (32.4)**	**1,945 (42.3)**	**1.53**	**1.30-1.79**	**<0.0001**

	Within 90-Days of THA				
	No Protrusio n (%)	Protrusio n (%)	OR	95% CI	*P* value
Hospital readmission	402 (9.2)	523 (11.4)	1.28	0.99-1.65	0.063
Postoperative dislocation	43 (1.0)	47 (1.0)	1.04	0.52-2.10	0.904
Periprosthetic fracture	**21 (0.5)**	**55 (1.2)**	**2.56**	**1.10-5.97**	**0.03**
Any revision surgery	78 (1.8)	132 (2.9)	1.65	0.96-2.80	0.067
Revision surgery for instability	26 (0.6)	39 (0.9)	1.46	0.61-3.51	0.4
Revision surgery for periprosthetic fracture	**16 (0.4)**	**51 (1.1)**	**3.02**	**1.18-7.78**	**0.022**
Revision surgery for infection	11 (0.3)	30 (0.7)	2.54	0.75-8.52	0.132

CI = confidence interval, OR = odds ratio, THA, total hip arthroplasty

a Zero patients in no protrusio and protrusio groups. Statistically significant (p<0.05) for bolded entries.

Although no statistically notable difference was observed between the 2 groups for risk of readmission, postoperative dislocation, postoperative infection, revision surgery for instability, or revision surgery for infection, patients with AP who underwent THA had an increased risk of periprosthetic fracture (1.2% versus 0.5%, OR = 2.56, 95% CI: 1.10 to 5.97, *P* = 0.03) and having to undergo revision surgery for periprosthetic fracture (1.1% versus 0.4%, OR = 3.02, 95% CI: 1.18 to 7.78, *P* = 0.022) than patients without AP within 90 days of index surgery (Table 5).

Finally, as compared to patients without AP, patients with AP had an increased average LOS (5.0 versus 4.3 days, *P* = 0.002) and cost ($27,736.30 versus $19,211.88, *P* < 0.0001) during their index hospitalization (Table 6).

**Table 6:** Index Hospitalization Length of Stay and Cost

	Length of Stay (d)			Cost ($)		
	Mean	95% CI	*P* value^a^	Mean	95% CI	*P* value^a^
No protrusio	4.3	4.1-4.6	—	19,211.88	18,511.83-19,911.94	—
Protrusio	5.0	4.7-5.3	0.002	27,736.30	20,946.62-22,525.99	<0.0001

CI = confidence interval

a *P*-value when compared with no protrusio group.

## Discussion

Acetabular deficiency with intrapelvic displacement of the acetabulum and femoral head, or AP, is a relatively rare finding, but one that is important to recognize because it can complicate THA surgery and outcomes. The purpose of this retrospective cohort study was to utilize a large, national database to compare outcomes after THA in patients with AP with those in patients without AP. To the authors' knowledge, this is the largest study, to date, evaluating the outcomes of patients with AP undergoing THA.

This study found that patients with AP undergoing THA tended to be older, were predominantly women, and had more medical comorbidities than patients without AP. The female predominance is likely multifactorial but may in part be due to increased rates of osteoporosis in women in addition to anatomic differences, including a wider pelvis that is subject to greater joint reaction forces and thus more prone to femoral head medialization.^4^ The higher prevalence of RA in women, as well as more robust RA disease activity, could also contribute to this female predominance.^25^ The conditions that predispose to the development of AP are for the most part also systemic conditions that affect other organ systems. Thus, it is expected that the development of AP is accompanied by a decline in overall health as patients age. In addition, these underlying conditions, as well as medications used in the treatment of these conditions, predispose patients to osteopenia,^25,26^ which is a known risk factor for the development of AP.

This study also found that patients with AP were more likely to require bone grafting during THA than patients without AP. Bone graft can be used to compensate for insufficient acetabular bone stock by augmenting the medial acetabular wall, thus preventing medialization of the hip center of rotation.^8^ Studies have shown the importance of restoring the hip center of rotation in patients with AP. Baghdadi and Larson et al^7^ demonstrated a 24% increased risk of aseptic cup revision in patients with AP for every 1 mm of medial or lateral distance away from the native hip center to the prosthetic head center. Despite some reported shortcomings of bone graft,^17,27,28^ most of the current literature supports its use to enhance biomechanical stability and improve clinical outcomes in patients with severe acetabular bone stock insufficiency.^5,10,12,29,30,31,32,33^

This study also revealed an increased need for blood transfusion in patients with AP undergoing THA, even after matching for patient age and ECI. The 28.5% incidence of blood transfusion in patients with AP is consistent with previous literature. Lorentz et al^34^ also showed an increased need for transfusion in patients with AP, with a transfusion incidence of 24.4%. The increased complexity of these operations often requires larger surgical approaches and longer operative time.^10,35^ It is therefore somewhat expected that these patients more often require blood transfusion. Given that this study has also shown that patients with AP are older and have more medical comorbidities than patients without AP, they are less likely to tolerate anemia. Therefore, it is important that surgeons be cognizant of blood loss and are prepared for this greater likelihood of transfusion.

Of the postoperative complications assessed, cardiac complications were the only subset that showed a notably higher incidence in the protrusio population. This is likely in part related to the increased incidence of blood transfusions in patients with AP as previously discussed, although a direct correlation also exists between inflammatory conditions and cardiovascular morbidity and mortality.^36^ The cardiovascular mortality rate is 45% higher in patients with inflammatory arthritis compared with the general population. This may be to direct inflammatory myocardial involvement, which can lead to fibrotic and electrophysiologic complications.^36,37^ Although cardiac complications were controlled in this study by matching for ECI and RA, the increased risk for cardiac complications in the AP population is important to be aware of before proceeding with THA. Comparison after propensity score matching also revealed no increased risk of death in patients with AP as compared to patients without AP. This echoes findings from previous studies, which have shown satisfactory outcomes in this patient population.^11,12,13,14,15,16^

**Figure 3:**
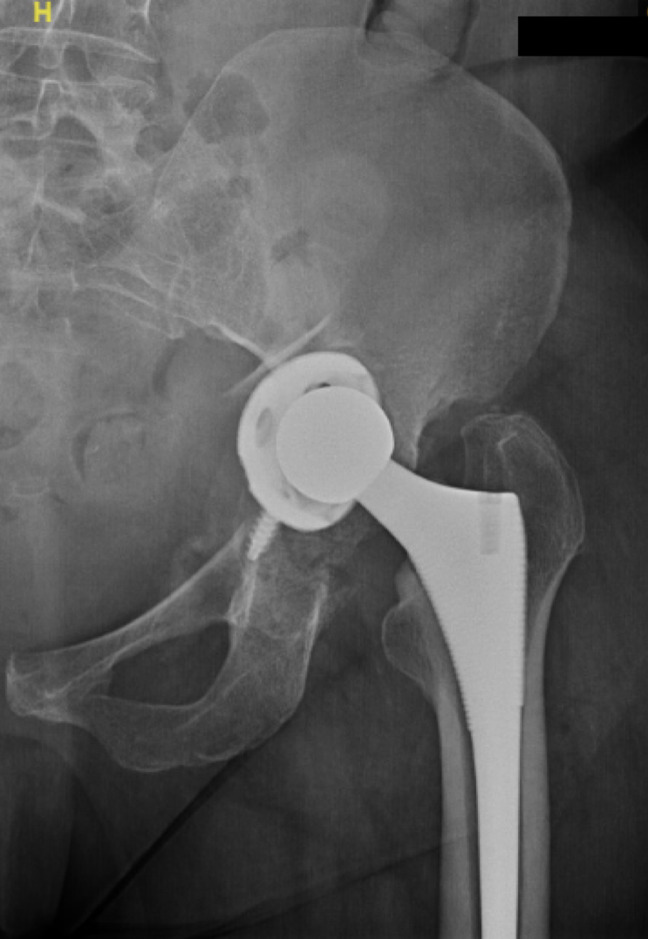
Postoperative radiograph of a patient with acetabular protrusio who underwent total hip arthroplasty complicated by a periprosthetic fracture of the acetabulum.

Previous literature has shown increased rates of intraoperative fractures in patients with AP who undergo THA.^10,38,39,40^ However, this is the first study to the authors' knowledge that has demonstrated an increase incidence of periprosthetic fracture occurring within 90 days of THA in patients with AP. This may be explained by a deficient medial wall characteristically seen in patients with AP that is prone to fracture during acetabular preparation and component impaction. Native AP hips are also difficult to dislocate during THA and sometimes are even completely autofused, which makes dislocation very challenging and may require an in situ neck cut before removal of the femoral head (Figure 1, A). This may also put patients with AP at increased fracture risk that may or may not be detected intraoperatively^25,26,41,42^ (Figure 3).

The statistically notable increased risk of periprosthetic fractures in this patient population warrants thorough consideration throughout the preoperative, intraoperative, and postoperative periods. Bone integrity should be assessed preoperatively with up-to-date imaging, and low bone mineral density should be appropriately treated before proceeding with surgery.^38^ Orthopaedic surgeons should communicate with internists and rheumatologists to determine whether any medications that could weaken bone integrity can be safely weaned or tapered before surgery. Postoperative rehabilitation should focus on fall prevention, and additional weight-bearing precautions should be considered on a case-by-case basis.

Regarding healthcare resource utilization, this study found a notably higher index hospitalization LOS and cost for patients with AP undergoing THA. It is plausible that the higher cost may be related to the variety of specialized implants used in the AP population.^7^ Comprehensive literature exists demonstrating the high healthcare resource expenditure associated with a longer index hospitalization LOS in patients who undergo THA.^42,43^ More importantly, accurate prediction of a longer LOS can minimize overall cost.^42^ It is therefore beneficial to manage expectations regarding LOS in this patient population, both for the individual patients themselves and for the healthcare system as a whole.

The authors recognize that this study has several limitations, starting with the inherent weakness of a retrospective database study and the potential for errors in coding entry, which could result in insufficient data capture. As cases of AP that underwent THA may not have been coded as such, the incidence of AP presented here likely underrepresents the true incidence. Furthermore, the various conditions associated with the development of AP may influence clinical outcomes and therefore confound results. The authors attempted to account for these differences by matching patient ECI and for a diagnosis of RA. Regardless of the underlying condition, AP can act as a marker for a patient at risk of increased cost and LOS, and therefore, these findings are still important to note because they can aid in managing expectations perioperatively. Relying on the NRD does not allow for the analysis of severity of acetabular deficiency, surgical time, surgical approach, and choice of implants, which are important factors in the management of patients with AP. Although this study was able to identify an increased incidence of periprosthetic fracture in patients with AP, it is unable to specify where these fractures occur (acetabular or femoral side), which would be helpful in analyzing why this risk is increased and how it can be mitigated. Finally, this study lacks patient-reported outcomes, leading to an inability to comment on subjective clinical improvement after THA in this patient population.

Despite these limitations, this study is the first to leverage the strength of a large, national database and reports on the largest cohort of patients with AP undergoing THA. Given the anatomical complexities of patients with AP, surgeons should approach THA knowing a higher likelihood of bone graft utilization and blood transfusion exists. In addition, special attention should be given postoperatively to the prevention of periprosthetic fractures, which may include measures to prevent falls and enhance bone mineral density in this vulnerable patient population. This study highlights the importance of recognizing AP in patients scheduled to undergo THA because these patients require careful preoperative planning and close postoperative monitoring.
